# Rapid and sensitive detection of *Mycoplasma synoviae* by an insulated isothermal polymerase chain reaction-based assay on a field-deployable device

**DOI:** 10.3382/ps/pew228

**Published:** 2016-07-07

**Authors:** Hung-Chih Kuo, Dan-Yuan Lo, Chiou-Lin Chen, Yun-Long Tsai, Jia-Fong Ping, Chien-Hsien Lee, Pei-Yu Alison Lee, Hsiao-Fen Grace Chang

**Affiliations:** * Yunlin-Chiayi-Tainan of Animal Disease Diagnostic Center, Department of Veterinary Medicine, National Chiayi University, Chiayi, Taiwan; † GeneReach Biotech, Taichung, Taiwan

**Keywords:** *Mycoplasma synoviae*, insulated isothermal PCR, point-of-need detection

## Abstract

*Mycoplasma synoviae* (MS), causing respiratory diseases, arthritis, and eggshell apex abnormalities in avian species, is an important pathogen in the poultry industry. Implementation of a biosecurity plan is important in MS infection management. Working on a field-deployable POCKIT™ device, an insulated isothermal polymerase chain reaction (iiPCR) assay has a potential for timely MS detection on the farm. The MS iiPCR assay had limit of detection 95% of about 9 genome equivalents by testing serial dilutions of a standard DNA. The detection endpoint of the assay for detection of MS genomic DNA was comparable to a reference real-time PCR. The assay did not crossreact with other important avian pathogens, including avian reovirus, *Mycoplasma gallisepticum, Staphylococcus aureus*, *Escherichia coli*, *Pasteurella multocida*, and *Salmonella* Pullorum. When 92 synovial fluid and respiratory tract swab samples collected from chickens, turkeys, and geese suspected of MS infection were tested, the clinical performance of the MS iiPCR had 97.8% agreement (Cohen's kappa value, 0.95) with that of the reference real-time PCR. In conclusion, the MS iiPCR/POCKIT™ system, working with field-deployable manual or automatic nucleic acid extraction methods, has potential to serve as a rapid and sensitive on-site tool to facilitate timely detection of MS.

## INTRODUCTION

*Mycoplasma synoviae* (**MS**), one of the important *Mycoplasma* species that infect avian species, has been listed as a notifiable mycoplasma by World Organization for Animal Health (**OIE**, http://www.oie.int/) (OIE, 2015). MS infection could lead to symptoms including sinusitis, airsacculitis, synovitis, and eggshell apex abnormalities and result in reduction in egg production and meat quality, leading to considerable economic losses in the poultry industry worldwide (Kleven, 2008; Landman, 2014). MS infection is found commonly in chickens, turkeys, and guinea fowl, but less frequently in ducks, geese, and pigeons (Kleven, 2008; OIE, 2015). The morbidity of about 5 to 15% in chicken and 1 to 20% in turkey has been associated directly with MS infection alone and indirectly through synergistic effects of co-infection with other pathogens (Raviv et al., 2007; Kleven, 2008).

Transmission of MS is accomplished laterally via direct contact and respiratory aerosols, and vertically within eggs. Like other pathogenic *Mycoplasmas*, MS could colonize and persist in the host (Noormohammadi, 2007; Kleven, 2008). Since MS infection may be asymptomatic and easily overlooked, routine MS monitoring has been recommended for biosecurity to help reduce MS outbreaks in the poultry industry (Landman, 2014). Furthermore, the symptoms caused by MS infection could not be differentiated easily from those caused by other avian pathogens, such as avian reovirus, *Mycoplasma gallisepticum* (**MG**), *Staphylococcus aureus*, *Escherichia coli*, *Pasteurella multocida*, and *Salmonella* spp., which in poultry species could cause symptoms similar to MS infection (Kleven, 2008; OIE, 2015). A live vaccine based on the temperature-sensitive MS-H strain is commercially available only in several countries (Morrow et al., 1998; Kreizinger et al., 2015). Therefore, keeping a flock MS-free through efficient biosecurity management has been suggested to be the most effective method to help protect the poultry investment (Kleven, 2008; Landman, 2014). Differentiation of MS from other pathogens could help implementation of proper therapeutic and/or prevention measures to improve flock health management. A rapid method to detect MS sensitively and specifically to help the diagnosis and monitoring of MS infection in the field is thus much needed (Kleven, 2008; Landman, 2014).

Currently, the methods recommended by OIE for MS detection are bacterial isolation, serological assays, and polymerase chain reaction (**PCR**) (OIE, 2015). MS isolation, the gold-standard method for MS detection, is a slow process and may take up to 28 d to complete (Feberwee et al., 2005). MS isolation may also be compromised by competition from other pathogens, especially in the cases of chronically infected animals, which in general have relatively low MS loads (Ewing et al., 1996; Kleven, 2008; Roussan et al., 2015). Serological tests, being less time-consuming than MS isolation, have been the most commonly used method for MS detection. Nevertheless, antibody development takes about 2 to 4 wk and the antibody levels in turkeys are relatively low (Kleven, 2008). Moreover, serological tests have relatively high risks of cross-reactivity (particularly with other *Mycoplasmas* spp.) and low sensitivity (Yoder, 1989; Ewing et al., 1996; Ewing et al., 1998; Kleven, 2008). Alternatively, being relatively rapid, specific, and sensitive, PCR has been accepted to detect MS in clinical samples (Hess et al., 2007; Sprygin et al., 2010; OIE, 2015). Analysis of the *vlhA* (hemagglutinin) gene allowed intraspecific sequence typing of MS in conventional PCR assays (Hong et al., 2004; Hammond et al., 2009; OIE, 2015). In addition, real-time PCR assays targeting the 16S rRNA or *vlhA* gene are available to differentiate MS from its close relatives, such as MG (Jarquin et al., 2009; Sprygin et al., 2010; Fraga et al., 2013; Huang et al., 2015). Compared to conventional PCR, real-time PCR has higher detection sensitivity and specificity and is easier and faster to perform. However, as it requires a specialized laboratory and well-trained technicians, real-time PCR has been used mainly in diagnostic laboratories. Therefore, a sensitive, specific, and user-friendly detection tool is still much needed for rapid MS detection in the field to facilitate timely diagnosis and monitoring of MS infection for the poultry industry.

A fluorescent probe hydrolysis insulated isothermal PCR (**iiPCR**) methodology has been developed specifically for on-site applications (Tsai et al., 2012). The reaction worked on a portable POCKIT™ Nucleic Acid Analyzer (POCKIT™; GeneReach, Taichung, Taiwan), which detected and processed reaction signals automatically to generate easy final positive and negative readouts. Carried out in a capillary tube and driven by the Rayleigh-Bènard convention (Krishnan et al., 2002), the reaction could generate detectable signals in less than 1 h. The iiPCR/POCKIT™ system has been demonstrated to achieve specific and sensitive detection of several bacterial and viral infections of veterinary interest in companion animals, livestock animals, and aquaculture animals (Tsai et al., 2012; Tsen et al., 2013; Balasuriya et al., 2014; Tsai et al., 2014; Wilkes et al., 2014; Ambagala et al., 2015; Lung et al., 2015; Wilkes et al., 2015a,b). In addition, a nucleic acid extraction step is generally required before PCR to remove reaction inhibitors in the sample matrix (Schrader et al., 2012). The iiPCR/POCKIT™ system is promising for field application because field-deployable automatic and manual nucleic extraction methods have been available to provide samples suitable for the reaction.

Recently, an iiPCR assay (POCKIT™ *M. synoviae* Detection Kit, GeneReach) targeting the *vlhA* gene of MS has been available commercially for MS detection on the POCKIT™ device. In this study, analytical sensitivity and specificity of the assay were evaluated. Furthermore, clinical performance of the assay was verified by comparison with that of a previously published MS real-time PCR using clinical samples collected from chickens, turkeys, and geese suspected of MS infection.

## MATERIALS AND METHODS

### Microorganisms and Plasmids

MS (WVU 1853 [NCTC 10124] strain; ATCC 25204), MG (NCTC 10115, PG 31, X95 strain, ATCC 19610), *Salmonella enterica* subsp. *enterica* serovar Pullorum (NRRL B-663 [52-16T, CDC 2490-60, R. Hugh 529] strain, ATCC 13036), *S. aureus* Rosenbach (Seattle 1945 strain, ATCC 25923), *E. coli* (CDC EDL 932 strain, ATCC 43894), *P. multocida* subsp. *multocida* (NCTC 10322 [W-9217] strain, ATCC 43137), and avian reovirus (ATCC VR-2449) were used in the analysis; the specificity assays were purchased from the American Type Culture Collection (Manassas, VA).

The pMSvlha and pMGvlha plasmids contained a fragment of the MS *vlhA* gene (nt 292195 - 292694, GenBank accession number AE017245) and a fragment of the v*lhA* gene from MG (pMGA1.7 precursor or hemagglutinin gene; nt 2901 - 3101, GenBank accession number U90714), respectively. They were synthesized on the basis of the pUC57 plasmid (Shanghai Generay Biotech, Shanghai, China). Concentrations of the plasmids were determined by a NanoDrop 2000 (Thermo Scientific, Wilmington, DE).

### Clinical Samples

A total of 92 samples, including 15 synovial fluids, 32 laryngeal swabs, 39 nasal swabs, and 6 tracheal swabs, were analyzed. The clinical samples were collected from chickens, turkeys, or geese suspected of MS infection at various poultry farms in Taiwan in 2014. The clinical signs included a greenish discoloration of dropping, pale comb, lameness, swelling joints, listlessness, dehydration, emaciation, rales, and sinusitis. The swabs were placed into 1 mL of minimal essential medium. Synovial fluid was collected by direct penetration of the joint space using a 23 to 25 G needle with a small (3 mL) syringe (Campbell, 1994). Samples were stored at –80°C before DNA extraction.

### Nucleic Acid Extraction

The taco™ mini Nucleic Acid Automatic Extraction System (taco™ mini; GeneReach) is a field-deployable device and can work with the iiPCR assay for on-site pathogen detection. In this study, nucleic acids were isolated by the taco™ mini device using the taco™ DNA/RNA Extraction Kit (GeneReach) according to the manufacturer's instructions. In brief, the wells of the extraction plate were filled with the designated reagent buffers. After 200 μL of the samples were added into the sample wells, the plate was loaded into a taco™ mini device for automatic DNA extraction. The DNA extracts eluted in 200 μL ELUTION BUFFER were individually collected and stored at –80°C for later use.

### M. synoviae Insulated Isothermal PCR

The MS iiPCR assay (POCKIT™ *M. synoviae* Detection Kit, GeneReach) designed to target the *vlhA* (hemagglutinin) gene of MS was provided in a lyophilized format. The reagent was reconstituted with 50 μL of Premix Buffer B right before use. After the addition of 5 μL of the nucleic acid sample, the reaction mixture was transferred to an R-tube™ (GeneReach), which was placed subsequently into a POCKIT™ device to complete the amplification, detection, and data interpretation steps. Positive/negative qualitative readouts were displayed on the screen of the device within 1 h.

### M. synoviae Real-Time PCR

The reference MS real-time PCR targeting the *vlhA* gene (Sprygin et al., 2010) was performed on an Applied Biosystems^®^ 7500 Real-Time PCR System (Thermo Fisher Scientific, Carlsbad, CA). The 25-μL reaction mixture contained 1 x PCR Reaction Buffer (BioMi, Taichung, Taiwan), 0.5 mM dNTP, 0.8 μM forward primer (5^′^-CCAGGAGGTGGTACAGTTGAC-3^′^), 0.8 μM reverse primer (5^′^-TTAATGCTTCTTTAACT(G/A)AATCTGA-3^′^), 0.2 μM probe (FAM-5^′^-CTGCTAAAACAGAAGCTAAAAC(C/T)GCTAT-3^′^-BHQ1), 30 nM ROX, and 2.5 units of Taq DNA polymerase (BioMi) and 2 μL DNA sample. The cycling program included one cycle at 95°C for 5 min, followed by 40 cycles of 95°C for 25 sec, 58°C for 35 sec, and 72°C for 45 sec. The amplicon was 124 base pairs long. A typical example of the MS real-time PCR (data not shown) had a linearity range between 10^6^ and 10^1^ copies of the standard plasmid DNA (pMSvlha) with a slope of –3.10. The correlation coefficient was 0.99 with a y-intercept of 36.5. All reactions that had a recorded threshold cycle (**C_t_**) number value were considered positive.

### Analytical Sensitivity and Specificity

Replicates of reactions containing different copies of the plasmid (100, 50, 20, 10, 5, and 0 copies) and serial dilutions of the MS genomic DNA (10-, 10^2^-, 10^3^-, 10^4^-, and 10^5^-fold) were used to determine the analytical sensitivity of the MS iiPCR assay. Dilutions of the pMSvlha plasmid or genomic DNA of a MS culture were prepared in 40 ng/μL yeast tRNA. For the evaluation of analytical specificity, nucleic acids of the organisms in the exclusivity panel and the pMGvlha (10^6^ copies) were tested.

### Statistical Analysis

Limit of detection 95% (LOD95%) of the assay was determined by probit analysis at 95% confidence interval by using the SPSS v14 (SPSS, Chicago, IL). Kappa analysis of a 2 × 2 contingency table was used to assess the agreement between 2 assays.

## RESULTS

### Analytic Sensitivity of M. synoviae Insulated Isothermal PCR Assay

To evaluate the sensitivity of the MS iiPCR assay on the POCKIT™ device, serial dilutions of the standard plasmid were tested. All reactions containing more than 20 copies of the template were positive (Table 1), while 90% (18/20) of the 10-copy and 95% (19/20) of the 5-copy reactions were positive. The LOD95% of the assay calculated by the probit regression analysis was 9 genome equivalents per reaction.

**Table 1.** Analytical sensitivity of MS iiPCR assay.

Plasmid DNA (copy)	No. total	No. positive	Rate (%)
100	10	10	100
50	20	20	100
20	20	20	100
10	20	18	90
5	20	19	95
0	24	0	0

In addition, the analytical sensitivity of the MS iiPCR assay for MS genomic DNA was compared to that of the published MS real-time PCR, which also targeted the *vlhA* gene (Sprygin et al., 2010). The detection limits were found at the 10^3^-fold dilution in both assays (Table 2), indicating that both assays had comparable analytical detection sensitivity for MS genomic DNA.

**Table 2.** Detection limit of MS iiPCR assay: Comparison with the reference real-time PCR.

Delution fold	Real-time PCR (C_t_)			iiPCR		
10	28.32	28.76	29.09	+	+	+
10^2^	31.97	32.32	32.24	+	+	+
10^3^	36.00	35.83	34.92	+	+	+
10^4^	ND	38.09	38.40	+	+	−
10^5^	ND	ND	ND	−	−	−

C_t_, threshold cycle; ND, not detected.

### Analytical Specificity of M. synoviae Insulated Isothermal PCR Assay

Analytical specificity of the MS iiPCR assay on the POCKIT™ device was verified with an exclusivity panel of 6 avian pathogens (MG, *S. enterica, S. aureus*, *E. coli*, *P. multocida*, and avian reovirus), which cause symptoms similar to those of the MS infection in poultry species (Jones, 2000; Kleven, 2008). The assay did not detect any of the 6 pathogens (data not shown). Furthermore, as the MG pMGA1.7 gene had been found to have >90% identity with the MS *vlhA* gene (Noormohammadi et al., 1998), the specificity of the MS iiPCR was further verified analytically with an artificial template of the homologous MG pMGA1.7 sequence. Negative test results were obtained, demonstrating its high specificity for the MS *vlhA* sequence (data not shown).

### Diagnostic Performance of M. synoviae Insulated Isothermal PCR Assay

The diagnostic performance of the MS iiPCR/POCKIT™ assay to detect MS in avian clinical samples was compared to that of the reference real-time PCR (Sprygin et al., 2010). Among the 92 samples (15 synovial fluids, 32 laryngeal swabs, 39 nasal swabs, and 6 tracheal swabs) collected from chickens, turkeys, and geese suspected of MS infection (Table 3), 29 tested positive (C_t_ = 21.54 to 38.88) and 63 were negative by the real-time PCR. The MS iiPCR assay also reacted positively and negatively with 29 and 63 samples, respectively. One of the 29 MS real-time PCR-positive samples was negative by the MS iiPCR, and 1 of the 63 real-time PCR-negative samples was positive by the MS iiPCR assay. The agreement between the 2 methods was 97.8% (95% confidence interval, 93.78 to 100%) with a Cohen's kappa value of 0.95 (Table 4), suggesting that the MS iiPCR assay on POCKIT™ system was comparable to the real-time PCR to detect MS in avian clinical samples.

**Table 3.** Detection of MS by MS real-time PCR and MS iiPCR assay: testing synovial fluid, and laryngeal, nasal, and tracheal swab samples from chickens, turkeys, and geese suspected of MS infection.

Sample type	Sample ID	Real-time PCR (C_t_)	iiPCR
Chicken/synovial fluid	CJ1	ND	−
	CJ2	ND	−
	CJ3	28.64	+
	CJ4	31.58	+
	CJ5	21.54	+
	CJ6	23.08	+
	CJ7	24.18	+
	CJ8	ND	+
	CJ9	ND	−
	CJ10	ND	−
	CJ11	ND	−
	CJ12	ND	−
	CJ13	ND	−
	CJ14	ND	−
	CJ15	ND	−
Chicken/swab	CL1	30.67	+
	CL2	36.75	+
	CL3	28.99	+
	CL4	33.67	+
	CL5	38.11	−
	CL6	36.11	+
	CL7	ND	−
	CL8	ND	−
	CL9	ND	−
	CL10	36.08	+
	CL11	35.67	+
	CL12	ND	−
	CL13	ND	−
	CL14	ND	−
	CL15	33.07	+
	CL16	ND	−
	CN1	38.88	+
	CN2	27.86	+
	CN3	33.10	+
	CN4	27.62	+
	CN5	ND	−
	CN6	ND	−
	CN7	ND	−
	CN8	ND	−
	CN9	ND	−
	CN10	ND	−
	CN11	35.24	+
	CN12	ND	−
	CN13	ND	−
	CN14	ND	−
	CN15	35.33	+
	CN16	ND	−
	CN17	ND	−
	CN18	ND	−
	CN19	ND	−
	CN20	ND	−
	CN21	ND	−
	CN22	ND	−
	CT1	ND	−
	CT2	ND	−
	CT3	ND	−
	CT4	ND	−
	CT5	ND	−
	CT6	ND	−
Turkey/swab	TL1	29.27	+
	TL2	29.93	+
	TL3	30.68	+
	TL4	27.12	+
	TL5	26.21	+
	TL6	35.35	+
	TN1	ND	−
	TN2	30.28	+
	TN3	35.54	+
	TN4	ND	−
	TN5	33.06	+
	TN6	ND	−
Goose/swab	GL1	ND	−
	GL2	ND	−
	GL3	ND	−
	GL4	ND	−
	GL5	ND	−
	GL6	ND	−
	GL7	ND	−
	GL8	ND	−
	GL9	ND	−
	GL10	ND	−
	GN1	ND	−
	GN2	ND	−
	GN3	ND	−
	GN4	ND	−
	GN5	ND	−
	GN6	ND	−
	GN7	ND	−
	GN8	ND	−
	GN9	ND	−
	GN10	ND	−
	GN11	ND	−

CJ, chicken synovial/joint fluid; CL, chicken laryngeal swab; CN, chicken nasal swab; CT, chicken tracheal swab; TL, turkey laryngeal swab; TN, turkey nasal swab; GL, goose laryngeal swab; GN, goose nasal swab; C_t_, threshold cycle; ND, not detected.

**Table 4.** A 2 × 2 performance comparison between MS iiPCR and real-time PCR: detection of MS in clinical samples from animals suspected of MS infection.

		Real-time PCR	
		
		
		Positive	Negative
iiPCR	Positive	28	1
	Negative	1	62

## DISCUSSION

In this study, the MS iiPCR assay was shown to have excellent analytical sensitivity and specificity to detect MS nucleic acids. The MS *vlhA* gene, also the target sequence of several PCR assays verified previously to detect MS specifically (Sprygin et al., 2010; Huang et al., 2015), was highly conserved among all sequences (n = 180) available in the GenBank. Notably, no cross-reactivity was found with the MG organism as well as with the plasmid containing the highly homologous region from the MG pMGA1.7 gene in this study, demonstrating excellent specificity of the MS iiPCR assay. Specificity of the assay was shown further by the fact that no signals were detected with the 6 important avian pathogens tested in this study. Moreover, no sequences with significant homology to the MS *vlhA* gene was found from nucleotide BLAST analysis (http://www.ncbi.nlm.nih.gov/BLAST) of the GenBank database, which included complete genomes of 3 *Mycoplasma* and *Acholeplasma* species that were likely to be encountered in the chicken trachea (*M. gallinarum* [GenBank accession no. JHZE00000000], and *M. gallinaceum* [GenBank accession no. CP011021], and *Acholeplasma laidlawii* [GenBank accession no. NC_010163]), suggesting that the MS iiPCR assay was not likely to have cross-reactivity with these species.

Test results of the 92 poultry samples from chickens, turkeys, and geese suspected of MS infection (Table 3) demonstrated that the MS iiPCR assay had clinical performance comparable to that of the reference real-time PCR (kappa = 0.95), which was demonstrated previously to have a detection limit for MS at 1 CFU equivalent/mL or 29 DNA copies per reaction (Sprygin et al., 2010). The one real-time PCR positive/iiPCR negative sample (CL5) had a C_t_ of 38.11, suggesting that the titers of MS in this sample were extremely low. Possible reasons for the one real-time PCR negative/iiPCR positive result include different effects of the sample matrix and the nucleic acid extraction method of the 2 PCR assays, as well as low MS titers in the sample. Overall, synovial fluids, laryngeal, nasal, and tracheal swab samples were shown to be suitable to work with the MS iiPCR assay for MS detection. In addition, the assay could be used for MS detection not only in chickens, but also in turkey and goose samples.

The iiPCR/POCKIT™ system, designed to be field-deployable to allow point-of-need use (Tsai et al., 2014), could be a potentially useful tool for on-site MS monitoring. The high specificity and sensitivity of the MS iiPCR assay shown in this report was facilitated by the TaqMan probe-based PCR amplification and detection technology. The MS iiPCR/POCKIT™ assay could be completed easily by a user with basic operation training because only several reaction assembly steps are needed before the reaction and no data interpretation afterwards was needed. The POCKIT™ device collected fluorescent signals before and after the reaction to calculated the signal-to-noise (S/N) ratio (Tsai et al., 2012). Based on the default thresholds, the default algorithms converted automatically the S/N ratio into “+” (positive), “–” (negative), or “?” (inconclusive) results which were shown on the screen. The “?” results indicated that the signals were ambiguous and the sample needed to be retested. Eight reactions could be completed per run in the compact device (28 × 25 × 8.5 cm, W × D × H; 2.1 kg). Besides being rugged, the device could operate on a rechargeable or car battery. The lyophilized reagents could be shipped to distant places without a cold chain and stay stable for up to 2 years in a refrigerator, eliminating the need to ship samples to a diagnostic laboratory. Thus, the relatively inexpensive iiPCR/POCKIT™ system could serve as a tool to greatly reduce test turn-around time and costs.

For on-site MS detection, the development of a loop-mediated isothermal amplification (**LAMP**)-based method targeting the MS *vlhA* gene was recently reported (Kursa et al., 2015). Relying on SYBR Green-based amplicon detection, the MS LAMP assay showed great analytical sensitivity and specificity. However, non-specific products may easily lead to false-positive results and the clinical performance of the MS LAMP assay remains to be verified.

Because the Taq DNA polymerase is sensitive to inhibitors present in different clinical samples (Schrader et al., 2012), it is often necessary to purify nucleic acids from samples before the PCR amplification step. In general, automatic nucleic acid extraction methods could reduce labor costs and improve performance consistency in PCR-based molecular diagnosis. Basic requirements for an easy nucleic acid extraction system to work at points of need include light weight, user-friendliness, and being car-battery chargeable. A field-deployable automatic nucleic acid extraction device (taco™ mini) that could fulfill these criteria is available to work with the iiPCR/POCKIT™ system. Combining the iiPCR/POCKIT™ system with the automatic extraction device, minimal hands-on time was required at the sample preparation step for taco™ mini and at the reaction assembly step for iiPCR. Although being less user-friendly than the automatic methods, manual nucleic acid extraction methods are usually cheaper and well accepted in situations where resources are limited. A manual nucleic acid extraction method (PetNAD™ Nucleic Acid Co-prep kit, GeneReach) is also available to prepare nucleic acids for the MS iiPCR assay. With both nucleic extraction methods, the time required to obtain results from samples could be within 2 h.

In conclusion, the rapid and user-friendly MS iiPCR/POCKIT™ system is a potential tool to help timely identification of MS infection at points of need in the poultry industry. With the high detection sensitivity and specificity demonstrated herein, the MS iiPCR reagent set could also be used to help diagnose MS infection at early and chronic stages.
